# LncRNA CASC9 promotes esophageal squamous cell carcinoma metastasis through upregulating LAMC2 expression by interacting with the CREB-binding protein

**DOI:** 10.1038/s41418-018-0084-9

**Published:** 2018-03-06

**Authors:** Yan Liang, Xuedan Chen, Yuanyuan Wu, Juan Li, Shixin Zhang, Kai Wang, Xingying Guan, Kang Yang, Yun Bai

**Affiliations:** 1 Department of Medical Genetics, College of Basic Medical Science, Third Military Medical University, Gaotanyan Street, Shapingba District, Chongqing, China; 2 Department of Cardiothoracic Surgery, Southwest Hospital, Third Military Medical University, Gaotanyan Street, Shapingba District, Chongqing, China

## Abstract

Esophageal squamous cell carcinoma (ESCC) is the main subtype of esophageal cancer. Long noncoding RNAs (lncRNAs) are thought to play a critical role in cancer development. Recently, lncRNA CASC9 was shown to be dysregulated in many cancer types, but the mechanisms whereby this occurs remain largely unknown. In this study, we found that CASC9 was significantly upregulated in ESCC tissues, with further analysis revealing that elevated CASC9 expression was associated with ESCC prognosis and metastasis. Furthermore, we found that CASC9 knockdown significantly repressed ESCC migration and invasion in vitro and metastasis in nude mice in vivo. A microarray analysis and mechanical experiments indicated that CASC9 preferentially affected gene expression linked to ECM–integrin interactions, including LAMC2, an upstream inducer of the integrin pathway. We demonstrated that LAMC2 was consistently upregulated in ESCC and promoted ESCC metastasis. LAMC2 overexpression partially compromised the decrease of cell migration and invasion capacity in CASC9 knockdowns. In addition, we found that both CASC9 and LAMC2 depletion reduced the phosphorylation of FAK, PI3K, and Akt, which are downstream effectors of the integrin pathway. Moreover, the reduction in phosphorylation caused by CASC9 depletion was rescued by LAMC2 overexpression, further confirming that CASC9 exerts a pro-metastatic role through LAMC2. Mechanistically, RNA pull-down and RNA-binding protein immunoprecipitation (RIP) assay indicated that CASC9 could bind with the transcriptional coactivator CREB-binding protein (CBP) in the nucleus. Chromatin immunoprecipitation (ChIP) assay additionally illustrated that CASC9 increased the enrichment of CBP and H3K27 acetylation in the LAMC2 promoter, thereby upregulating LAMC2 expression. In conclusion, we demonstrate that CASC9 upregulates LAMC2 expression by binding with CBP and modifying histone acetylation. Our research reveals the prognostic and pro-metastatic roles for CASC9 in ESCC, suggesting that CASC9 could serve as a biomarker for prognosis and a target for metastasis treatment.

## Introduction

Esophageal cancer (EC) is one of the most aggressive cancer types with an increasing incidence worldwide [1]. Esophageal squamous cell carcinoma (ESCC) is the predominant histological cancer type, accounting for ~80% of EC cancers [2]. Despite intensive clinical efforts using multiple therapeutic approaches, the overall 5-year survive rate remains at 15–25% due to a combination of both local invasion and distant metastasis [3]. Many patients with esophageal cancer already exhibit metastasis at diagnosis, even when tumors are superficial. Ensuing complications from tumor metastasis account for the majority of cancer-associated deaths [4]. Recently, many studies have revealed that alterations in gene expression, cytokine secretion, epithelial-to-mesenchymal transition (EMT), and the tumor microenvironment were associated with metastasis [5], which could only partially elucidate this complex process in a specific cancer type. Therefore, understanding molecular mechanisms of tumor metastasis are urgent and will provide new opportunities for tumor treatment.

The extracellular matrix (ECM) is a highly complex, fibrous network composed of a variety of biological molecules. Apart from providing structural integrity and scaffolding of tissue, the ECM directs a diverse set of functions through forming focal adhesions with the integrin family on cell membranes [6]. Epithelial tumor cells often secrete abundant amounts of laminin-332 (encoded by LAMA3, LAMB3, and LAMC2) and frequently express integrin α6β4 (encoded by ITGA6 and ITGB4). Laminin-332 is one of the main types of ECM molecules and the main ligand for integrin α6β4 [7]. Upon ligation, the integrin pathway is activated through phosphorylation of focal adhesion kinase (FAK), inducing downstream signaling pathways [8]. Indeed, FAK, PI3K, and Akt hyperactivation are commonly found in human malignancies, such as breast cancer, lung cancer, and ESCC, and are associated with tumor metastasis [9–11].

Long noncoding RNAs (lncRNAs) comprise a class of transcripts that are >200 nt in length and do not serve as templates for protein synthesis [12]. LncRNAs are classified into the following four broad categories according to their location relative to nearby protein-coding genes: antisense, bidirectional, intronic, and intergenic, with each category exhibiting distinct regulatory patterns [13]. Notably, lncRNA signatures reveal their involvement in tumorigenesis when aberrantly expressed [14–16], which is correlated with tumor behavior and prognosis. In esophageal cancer, lncRNAs MALAT1, HOTAIR, AFAP1-AS1, HNF1A-AS1, and CCAT1 are upregulated and promote cancer metastasis through multiple mechanisms, including miRNA sponging, epigenetic modification, and transcription regulation [17–21]. These findings suggest that lncRNAs may have utility as biomarkers for metastasis surveillance and may serve as therapeutic targets to inhibit metastasis.

In our previous study, we identified lncRNA cancer susceptibility candidate 9 (CASC9, NCBI), which was highly overexpressed in ESCC, could promote ESCC growth [22]. Simultaneously, several research groups revealed that CASC9 was also upregulated in many cancer types, including esophageal cancer, gastric cancer (GC), pancreatic ductal adenocarcinoma (PDAC), and nasopharyngeal carcinoma (NC) [23–26]. These researches indicate that CASC9 function as an oncogene in ESCC pregress, but the underlying mechanisms remain poorly understood. In this study, we demonstrated the pro-metastatic role of CASC9 in ESCC both in vitro and in vivo and that CASC9 regulated many genes related to ECM–integrin interaction. We also revealed that CASC9 upregulated LAMC2 expression through interacting with the CREB-binding protein (CBP), subsequently activating FAK-PI3K/Akt signaling pathways to promote metastasis.

## Results

### CASC9 overexpression in ESCC tissues correlates with ESCC aggressiveness

**Fig. 1:**
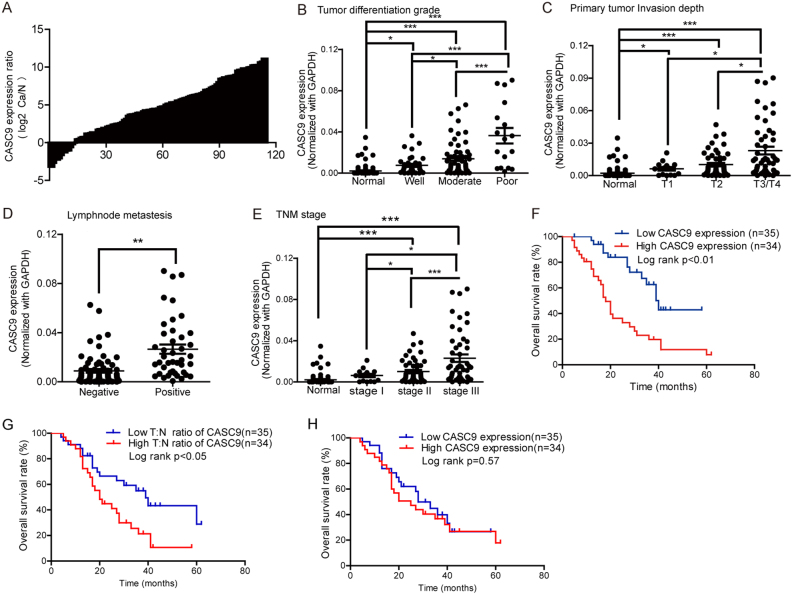
CASC9 overexpression in ESCC tissues correlates with ESCC aggressiveness. **a** Fold change in CASC9 expression between ESCC tissues and normal tissues normalized by log2 using RT-qPCR. **b** The correlation between CASC9 expression and tumor differentiation grade: normal, well, moderate, and poor. **c** The correlation between CASC9 expression and primary tumor invasion depth. T1: mucous layer, T2: muscularis propria layer, T3/T4: the adventitia layer and surrounding tissues. **d** The correlation between CASC9 expression and lymph node metastasis. Negative: no lymph node metastasis, positive: with lymph node metastasis. **e** The correlation between CASC9 expression and TNM stage. Stage I, stage II, and stage III represent different TNM stage of ESCC according to the latest AJCC (*American Journal of Critical Care*) guide. **f** High CASC9 expression in tumor tissues was associated with reduced survival of ESCC patients, as analyzed by Kaplan–Meier method. **g** High T:N expression ratio of CASC9 was associated with reduced survival. T:N, CASC9 expression ratio between tumor and adjacent normal tissue. **h** No significant association between CASC9 expression and survival was detected in normal tissues. *P < 0.05, **P < 0.01, ***P < 0.001

Our previous study demonstrated that CASC9 was overexpressed in ESCC tissues and correlated with ESCC growth and proliferation. To investigate the association between CASC9 and ESCC metastasis, we measured CASC9 expression in a larger cohort of paired ESCC tissues and adjacent normal tissues using reverse transcription and quantitative PCR (RT-qPCR). We found that CASC9 was overexpressed in 88.7% (102/115) of ESCC tissue compared to normal tissue samples (Fig. 1a). Further analysis revealed that tumor differentiation grade, primary tumor invasion depth, lymph node metastasis, and advanced TNM stage were all positively correlated with CASC9 expression (Fig. 1b, c, d, e), which strongly suggested that CASC9 might contribute to ESCC malignance, particularly metastasis. To confirm the CASC9 expression patterns, we divided the samples into a CASC9 low-expression group and CASC9 high-expression group according to the median CASC9 expression in ESCC tissues. As expected, primary tumor invasion depth, lymph node metastasis, and TNM stage were significantly different between the two groups (Table 1), while tumor differentiation grade showed no difference, likely due to lack of enough poorly differentiated samples. A prognostic analysis using 69 ESCC tissues with complete follow-up information revealed that both high CASC9 expression in ESCC tissues and high T:N ratio were associated with reduced overall survival (Fig. 1f, g), while no significant association between CASC9 expression in normal tissues and survival was detected (Fig. 1h). Therefore, we concluded that CASC9 overexpression was associated with ESCC aggressiveness and poor prognosis.

**Table 1:** Correlation between CASC9 expression and ESCC clinical parameters

Clinical parameters	Low expression	High expression	*P* value^a^
	*N* = 57	*N* = 58	
Age (years)			
<60	32	33	0.935
>60	25	25	
Gender			
Male	43	44	0.786
Female	14	14	
Differentiation grade			
Well/moderate	52	47	0.114
Poor	5	11	
Primary tumor invasion depth			
T1/T2	39	26	0.011*
T3/T4	18	32	
Lymph node metastasis			
Negative	47	27	<0.01**
Positive	10	31	
TNM stage			
I/II	50	35	0.001**
III/IV	7	23	
Smoking			
Yes	35	38	0.558
No	22	20	
Drinking			
Yes	34	37	0.648
No	23	21	

* *P* < 0.05; **P < 0.01

^a^ Chi-squared test results

We then investigated CASC9 expression in human ESCC cell lines (EC109, KYSE150, and KYSE450) and correspondent metastatic ability. Compared to the normal esophageal epithelial cell line Het-1A, we found that CASC9 expression was significantly higher in ESCC cell lines (Supplementary Figure 1A). In addition, the metastatic ability of ESCC cell lines enhanced as the expression of CASC9 increased (Supplementary Figure 1B), further demonstrating that CASC9 plays a critical role in ESCC metastasis.

### CASC9 promotes ESCC migration and invasion in vitro and metastasis in vivo

**Fig. 2:**
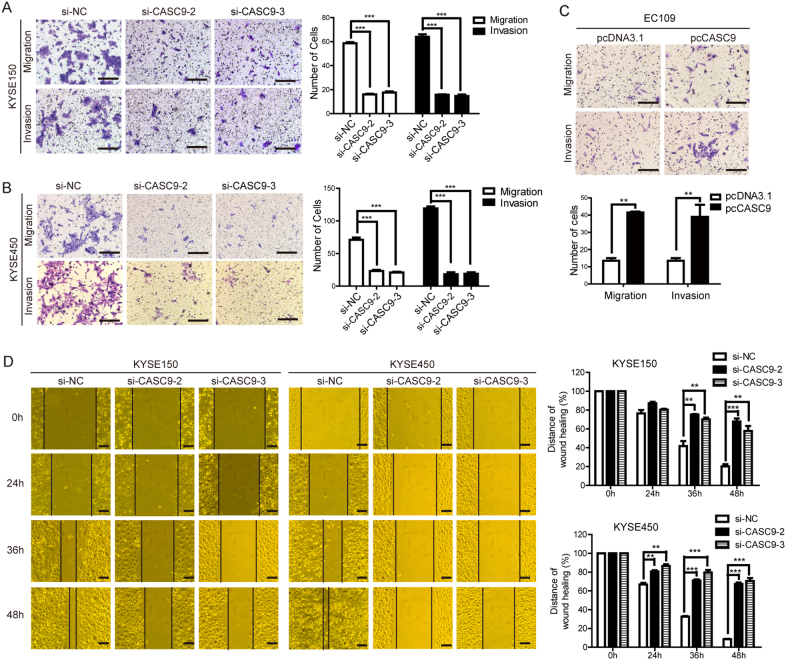
CASC9 promotes ESCC migration and invasion in vitro. **a**, **b** Transwell assays were performed in KYSE150 and KYSE450 cells transfected with two different siRNAs. The number of cells that migrated or invaded were counted in three different fields. Original magnification, ×200. Scale bars, 200 µm. **c** Transwell assays were performed in EC109 cells transfected with different vectors. Original magnification, ×200. Scale bars, 200 µm. **d** Wound healing assay in KYSE150 and KYSE450 cells transfected with two siRNAs. Original magnification, ×100. Scale bars, 200 µm. The distance of wound healing was measured and calculated as a percentage of the distance at 0 h. *P < 0.05, **P < 0.01, ***P < 0.001

To investigate the biological role of CASC9 in ESCC metastasis, we performed a knockdown of CASC9 expression in KYSE150 and KYSE450 cells using three siRNAs targeting different sites within CASC9. And si-CASC9-2 and si-CASC9-3, which had better knockdown efficiencies, were chosen to use in all subsequent experiments (Supplementary Figure 1C). Transwell assay demonstrated that CASC9 depletion almost completely inhibited the migration and invasion ability of KYSE150 and KYSE450 using two different siRNAs (Fig. 2a, b, respectively). Conversely, ectopic overexpression of CASC9 increased cell migration and invasion ability in EC109 cells (Fig. 2c). A wound healing assay consistently confirmed that CASC9 depletion inhibited cell motility (Fig. 2d). The reciprocal effects of knockdown and overexpression of CASC9 in vitro suggested that CASC9 facilitated ESCC metastasis.

**Fig. 3:**
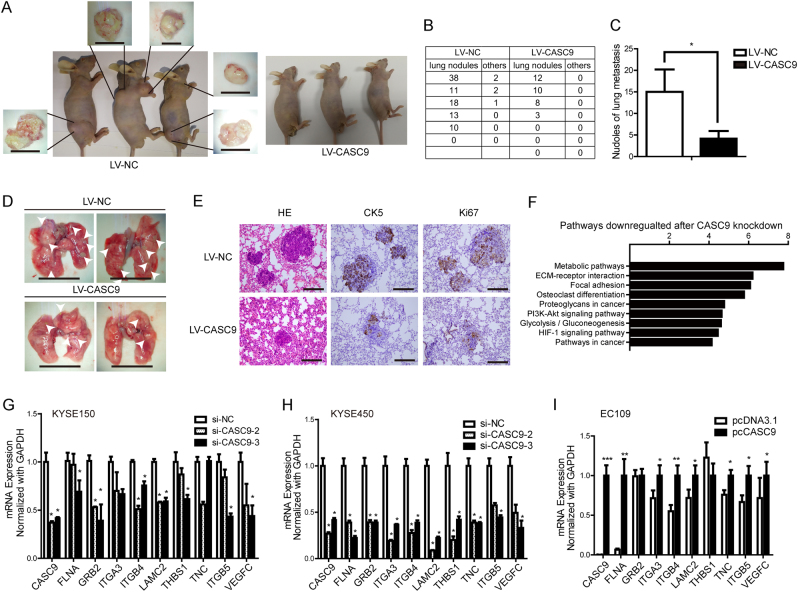
CASC9 promotes ESCC metastasis in vivo. **a** Representative images of mice from different treatment groups 8 weeks after tumor injections. Three mice from the LV-NC group presented bone and other remote metastasis. The metastatic tumors were detected under a stereoscope. Scale bars, 1 cm. **b** Number of metastatic nodules in lungs and other organs in mice from LV-CASC9 and LV-NC groups. **c** Bar chart of lung metastasis nodules in LV-CASC9 and LV-NC groups. **d** Representative images of lung metastases under stereoscope. Original magnification, ×1.5. Scale bars, 1 cm. **e** Representative images of HE and IHC staining of lung metastasis. Original magnification, ×400. Scale bars, 100 µm. **f** Signaling pathways of downregulated genes after CASC9 knockdown in KYSE450 cells detected by microarray analysis. **g**, **h**, **i** Gene expression of ECM–receptor interactions and focal adhesion pathways after CASC9 knockdown or overexpression detected by RT-qPCR. *P < 0.05, **P < 0.01, ***P < 0.001

To further confirm the metastasis promotion by CASC9 in vivo, KYSE150 cells stably transfected with LV-CASC9 and LV-NC were injected into the tail vein of BALB/c mice to establish a metastasis model. We found that knockdown of CASC9 resulted in significantly decreased metastasis to the lungs, bone, and other sites. Notably, three mice from the LV-NC group presented bone and other remote metastasis, while no mice from the LV-CASC9 group exhibited metastasis in other places (Fig. 3a). In addition, metastatic nodules at the lung surface in the LV-NC group were significantly more frequent than in the LV-CASC9 group (Fig. 3b, c). Metastatic nodules from LV-NC group were also much larger than that in the LV-CASC9 group (Fig. 3d). This phenomenon was further confirmed by examining the lung tissues using HE and IHC staining using CK5 antibodies (Fig. 3e). Bone and other metastasis sites were also further validated (Supplementary Figure 1D). IHC staining using Ki67 antibodies implied that CASC9 could facilitate the proliferation ability of metastatic tumor (Fig. 3e). Hence, both the in vitro and in vivo data supported the pro-metastatic role of CASC9 in ESCC.

### CASC9 upregulates the expression of genes associated with ECM–receptor interaction and focal adhesion pathways

To generate comprehensive insights into the molecular mechanisms underlying CASC9 function, we reanalyzed the microarray analysis we performed before, comparing KYSE450 cells transfected with either si-CASC9-2 or a negative control. A pathway analysis demonstrated that CASC9 knockdown had major consequences on genes that were primarily connected with metabolic pathways, ECM–receptor interactions and focal adhesions (Fig. 3f). The ECM–receptor interactions and focal adhesion pathways consist of many well-known genes functioning in cell motility and cancer metastasis, which is consistent with the pro-metastatic role of CASC9. Then, using CASC9 loss- and gain-of-function experiments, we confirmed that the genes identified by microarray decreased in CASC9 knockdown cells and increased in CASC9 overexpressing cells (Fig. 3g, h, i). These data indicated that CASC9 impacted expression of genes related to cancer metastasis.

**Fig. 4:**
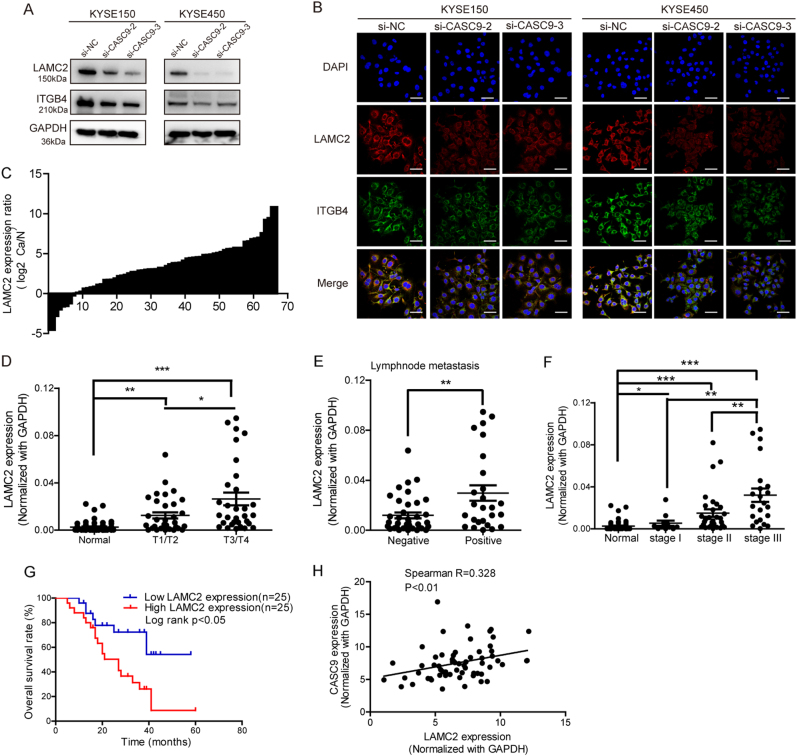
CASC9 upregulates the expression of genes associated with ECM–receptor interactions and focal adhesion pathways. **a**, **b** Western blot and IF indicates the expression of LAMC2 and ITGB4 after CASC9 knockdown using two siRNAs in KYSE150 and KYSE450 cells. Original magnification, ×400. Scale bars, 40 µm. **c** Fold change in LAMC2 expression between ESCC tissues and normal tissues normalized by log2 using RT-qPCR. **d** The correlation between LAMC2 expression and primary tumor invasion depth. T1/T2: mucous layer and muscularis propria layer, T3/T4: the adventitia layer and surrounding tissues. **e**, **f** ESCC patients with lymph node metastasis and advanced TNM stage showed higher LAMC2 expression. Negative: no lymph node metastasis, positive: with lymph node metastasis. **g** Kaplan–Meier analysis revealed that high LAMC2 expression predicted poor prognosis in ESCC. **h** Correlation analysis of LAMC2 and CASC9 expression in ESCC tissues by RT-qPCR. Axis values are transformed by −Log2 of CASC9 expression. *P < 0.05, **P < 0.01, ***P < 0.001

Among the most significantly differentially expressed genes, ITGA3, ITGB4, and ITGB5 are integrin-coding genes, and LAMC2 codes for the γ2 chain of laminin-332. All these molecules are upstream of the integrin pathway, so we speculated that CASC9 might regulate the integrin pathway through controlling ECM–integrin interactions. We examined ITGA3, ITGB4, ITGB5, and LAMC2 expression in ESCC cell lines and found that LAMC2 and ITGB4 were overexpressed in ESCC cell lines, especially LAMC2, while ITGA3 and ITGB5 were not (Supplementary Figure 1E, F). Next, using western blot and immunofluorescence, we validated the protein levels alteration of LAMC2 and ITGB4 in response to CASC9 knockdown (Fig. 4a, b). Since integrin α6β4 is the main integrin type distributed in the cell membrane of epithelial tissue and laminin-332 is the main ligand of α6β4, we hypothesized that CASC9 might regulate the integrin pathway by controlling LAMC2 or ITGB4 expression.

### CASC9 metastasis promotion is mediated by LAMC2

To investigate whether LAMC2 and ITGB4 were direct targets of CASC9, we studied the relationship between CASC9 and LAMC2/ITGB4 with respect to expression and function. Initially, we examined LAMC2 and ITGB4 expression and their association with clinical characteristics of ESCC tissues. We discovered that LAMC2 and ITGB4 protein expression were both higher in 80% (20/25) of ESCC tissues (Supplementary Figure 1G). Next, the RT-qPCR results indicated that LAMC2 was overexpressed in 89.4% (59/66) of ESCC tissues (Fig. 4c), and high LAMC2 expression was associated with primary tumor invasion depth, lymph node metastasis, and advanced TNM stage (Fig. 4d, e, f). These results were further confirmed by Table 2. More importantly, high LAMC2 expression predicted poor prognosis (Fig. 4g). These results are all consistent with the observed increased CASC9 in ESCC tissues, indicating that LAMC2 was likely a downstream target of CASC9. We further analyzed the correlation between CASC9 and LAMC2 expression in ESCC tissues and found a positive correlation, further supporting our presumption (Fig. 4h). The alterations of ITGB4 were similar to those of LAMC2, but they were not as significant as those of LAMC2 (Supplementary Figure 2A–E). Thus, subsequent experiments focused on whether CASC9 function was a result of LAMC2 expression regulation.

**Table 2:** Correlation between LAMC2 expression and ESCC clinical parameters

Clinical parameters	Low expression	High expression	*P* value^a^
	*N* = 32	*N* = 32	
Age (years)			
<60	12	17	0.209
>60	20	15	
Gender			
Male	23	30	0.02*
Female	9	2	
Differentiation grade			
Well/moderate	30	25	0.072
Poor	2	7	
Primary tumor invasion depth			
T1/T2	20	13	0.08
T3/T4	12	9	
Lymph node metastasis			
Negative	23	15	0.042*
Positive	9	17	
TNM stage			
I/II	26	16	0.008**
III/IV	6	16	
Smoking			
Yes	18	24	0.114
No	14	8	
Drinking			
Yes	21	24	0.412
No	11	8	

* *P* < 0.05; **P < 0.01

^a^ Chi-squared test results

**Fig. 5:**
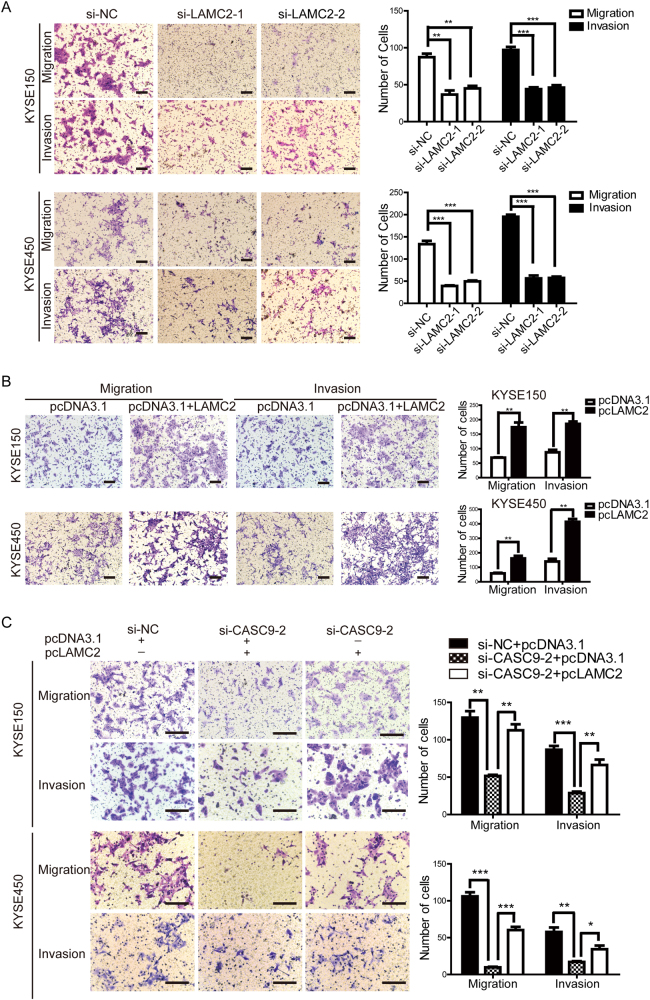
CASC9 promotes ESCC metastasis through regulation of LAMC2 expression. **a** Transwell assays showed that LAMC2 knockdown attenuated the migration and invasion abilities of KYSE150 and KYSE450 cells. Original magnification, ×100. Scale bars, 200 µm. **b** Transwell assays showed that LAMC2 overexpression enhanced the migration and invasion abilities of KYSE150 and KYSE450 cells. Original magnification, ×100. Scale bars, 200 µm. **c** The rescue experiment with Transwell assays was performed in KYSE150 and KYSE450 cells co-transfected with si-NC or si-CASC9-2 and pcDNA3.1 or pcLAMC2. Original magnification, ×100. Scale bars, 200 µm. *P < 0.05, **P < 0.01, ***P < 0.001

Because our results showed that CASC9 promoted ESCC metastasis, we next addressed the functional role of LAMC2 in conveying ESCC metastatic potential. The silencing of LAMC2 significantly attenuated the number of motile cells in KYSE150 and KYSE450 cells (Fig. 5a and Supplementary Figure 2F), and ectopic overexpression of LAMC2 enhanced cell migration and invasion ability in vitro (Fig. 5b and Supplementary Figure 2G). These findings provided evidence that LAMC2 played a similar role as CASC9 in orchestrating ESCC metastasis. Next, we performed rescue experiments to investigate whether CASC9 promoted ESCC metastasis through LAMC2. We ectopically overexpressed LAMC2 with simultaneous knockdown of CASC9 expression (Supplementary Figure 2H, I) and discovered that overexpression of LAMC2 partially attenuated the decreased cell migration and invasion capacity caused by CASC9 knockdown (Fig. 5c and Supplementary Figure 2J). Therefore, our findings indicated that LAMC2 was a major downstream mediator of CASC9-induced metastatic activity.

### CASC9 activates the FAK-PI3K/Akt signaling pathways through LAMC2

**Fig. 6:**
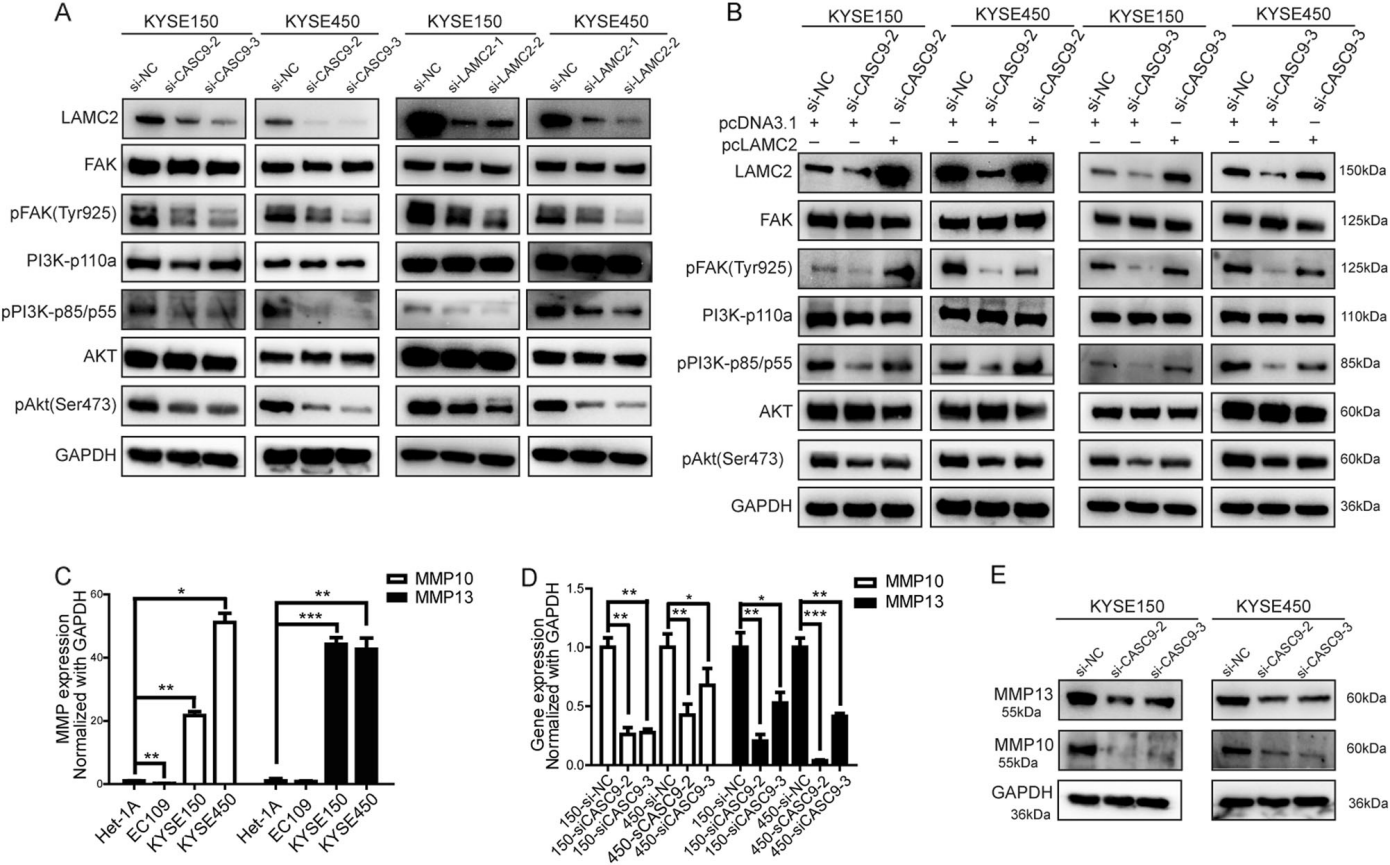
CASC9 activates FAK-PI3K/Akt signaling pathways through LAMC2. **a** Western blot analysis showed altered levels of pFAK, pPI3K, and pAkt after CASC9 or LAMC2 knockdown in KYSE150 and KYSE450 cells. **b** Western blot analysis of FAK-PI3K/Akt signaling pathways of the rescue experiment. **c** RT-qPCR analysis of MMP10 and MMP13 expression. **d**, **e** The expression of MMP10 and MMP13 in KYSE150 and KYSE450 cells after CASC9 knockdown. *P < 0.05, **P < 0.01, ***P < 0.001

There is evidence that integrin α6β4 binding to laminin-332 promotes tumor metastasis through focal adhesion kinase (FAK) [27, 28], whose phosphorylation induces the activation of downstream PI3K and Akt signaling pathways. As a result, we found that both CASC9 and LAMC2 depletion decreased pFAK, pPI3K, and pAkt levels, but they had no influence on total protein levels (Fig. 6a), which was in agreement with the pro-metastatic role of CASC9 and LAMC2. Then, rescue experiments showed that ectopic overexpression of LAMC2 reversed the decrease in pFAK, pPI3K, and pAkt levels caused by CASC9 depletion (Fig. 6b). In addition, we found that MMP10 and MMP13, functional molecules of the FAK-PI3K/Akt signaling pathways, were highly expressed in ESCC (Fig. 6c) and CASC9 depletion reduced MMP10 and MMP13 expression (Fig. 6d, e). All together, these results indicated that CASC9 could activate the FAK-PI3K/Akt signaling pathways through LAMC2 to promote ESCC metastasis.

### CASC9 upregulates LAMC2 expression by interacting with CBP

**Fig. 7:**
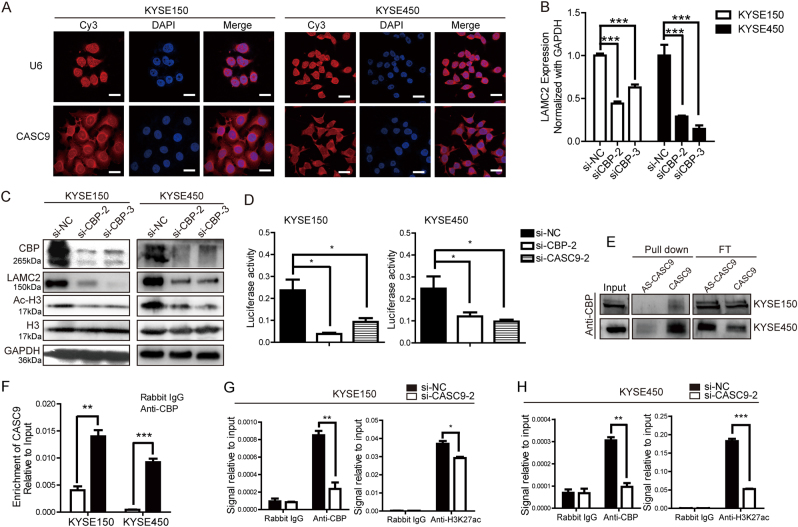
CASC9 upregulates LAMC2 expression through interacting with CBP. **a** Subcellular localization of CASC9 and U6 in KYSE150 and KYSE450 detected by RNA FISH. Original magnification, ×630. Scale bars, 20 µm. **b**, **c** RT-qPCR and western blot analysis of LAMC2 expression after CBP knockdown in KYSE150 and KYSE450. **d** Luciferase reporter assay was performed using vector-containing sequences of the LAMC2 promoter region. **e** RNA pull-down assay showed that CASC9 could retrieve CBP in KYSE150 and KYSE450 cells as detected by western blot. Pull-down indicates the retrieved protein. FT represents the flow-through protein after RNA–protein binding reaction. Input indicates the total cell protein used. **f** RIP experiment showed that anti-CBP antibody could precipitate CASC9 in KYSE150 and KYSE450 cells. **g**, **h** ChIP-qPCR revealed that CASC9 knockdown decreased the enrichment of CBP and the level of H3K27ac acetylation at the promoter region of LAMC2. *P < 0.05, **P < 0.01, ***P < 0.001

To determine the mechanism underlying CASC9’s regulation of LAMC2, we first performed an RNA FISH experiment to determine the cellular localization of CASC9. The result showed that CASC9 was distributed in both the cytoplasm and nucleus (Fig. 7a), indicating that CASC9 may function in the nucleus and/or cytoplasm and regulate gene expression at the transcriptional or epigenetic level. To explore these possibilities in more detail, we implemented bioinformatics analysis and found abundant H3K27ac signals in the promoter region of LAMC2 (Supplementary Figure 3A), suggesting that LAMC2 might be regulated by chromatin acetylation. We next treated ESCC cells with C646, a histone acetyltransferase (HAT) inhibitor, and found that LAMC2 expression was significantly decreased in response (Supplementary Figure 3B). CREB-binding protein (CBP) is one of the mostly important enzymes that modify chromatin acetylation, so we wondered if CBP might participate in LAMC2 regulation. In accordance to our assumption, decreasing the expression of CBP using siRNAs (Supplementary Figure 3C) significantly reduced the mRNA and protein levels of LAMC2 (Fig. 7b, c). Moreover, ChIP assay indicated that the promoter region of LAMC2 was enriched with CBP binding and H3K27ac signals (Supplementary Figure 3D). These data demonstrated that CBP could stimulate transcription of LAMC2 in ESCC cells through binding to the promoter and modifying histone acetylation.

Knowing that both CASC9 and CBP could upregulate LAMC2 expression at the mRNA and protein level, we next studied whether they participate in LAMC2 mRNA synthesis. A luciferase reporter assay showed that depletion of CASC9 or CBP attenuated the activity of the LAMC2 promoter, indicating that CASC9 and CBP could regulate LAMC2 expression at the transcriptional level (Fig. 7d). Then, to investigate the role of CASC9 during CBP regulation of LAMC2, we measured CBP expression after CASC9 knockdown; our results showed that CASC9 did not affect CBP expression (Supplementary Figure 3E), so we assumed that CASC9 might function through recruiting CBP to target genes. RNA–protein pull-down assays indicated that CASC9, but not antisense CASC9, distinctively retrieved CBP from KYSE150 and KYSE450 total protein extracts in vitro (Fig. 7e). Moreover, RIP assays confirmed that there was substantial enrichment of CASC9 using anti-CBP antibody compared to negative control, with U1 of SNRNP70 as a positive control (Fig. 7f and Supplementary Figure 3F). These findings strongly suggested that CASC9 could remarkably bind with CBP. Furthermore, ChIP assays indicated that CASC9 depletion decreased the enrichment of CBP and H3K27ac acetylation at the promoter region of LAMC2, but not the negative control, MyoD gene (Fig. 7g, h and Supplementary Figure 3G). Therefore, we concluded that CASC9 binding with CBP increased the enrichment of CBP and histone acetylation at the promoter, thus inducing the transcription of LAMC2.

## Discussion

This study highlights lncRNA CASC9’s function and mechanisms in regulating ESCC metastasis. CASC9 was originally identified and predicted to be a novel putative onco-lncRNA in ESCC [29, 30]. It was subsequently determined that CASC9 was overexpressed in many cancer types and promoted cell proliferation, differentiation, and invasion [23–26]. Previously, our work demonstrated that upregulation of CASC9 promoted ESCC growth and proliferation through regulation of PDCD4 expression [22]. In this study, we show that CASC9 was significantly upregulated in ESCC tissues, and high CASC9 expression correlated with a deeper primary invasion depth, positive lymph node metastasis, and advanced TNM stage, suggesting that CASC9 can be a strong predictor for ESCC metastasis and prognosis. In addition, we demonstrated that CASC9 promoted ESCC metastasis in vitro and in vivo, making CASC9 a potential target for metastasis inhibition in ESCC. In addition, we found that CASC9 promoted the proliferation of metastatic tumors in vivo, suggesting that CASC9 accelerated the terminal phase of ESCC metastasis. In our previous study, we demonstrated that CASC9 promoted proliferation of primary tumor [22]. Therefore, we conclude that CASC9 could increase the proliferation of both primary site and metastatic tumors. Taken together, CASC9 can be a promising potential biomarker for prognosis and therapeutic target in ESCC.

Our research demonstrated that CASC9 promotes ESCC metastasis through regulation of many genes related with ECM–integrin interactions. Researches demonstrate that imbalance of laminins such as expression disorder or genomic variation is thought to be correlated with tumorigenesis [31]. For example, there is ongoing research concerning the LAMC2 overexpression in various human malignancies, including lung cancer, gastric cancer, pancreatic cancer, and cutaneous squamous cell carcinoma (SCC) [32–37]. In particular, LAMC2 expression frequently has been identified at the invasive front of numerous epithelial cancers [38–41]. In this study, we identified LAMC2 as the most highly differentially expressed gene in response to CASC9 knockdown, and we observed that LAMC2 was upregulated in ESCC and correlated with poor clinical outcomes. In addition, LAMC2 facilitated ESCC invasion and metastasis, which was consistent with the pro-metastatic role of CASC9. Then, we described an intensive correlation between CASC9 and LAMC2 expression in ESCC tissues, plus that LAMC2 overexpression could partially attenuate the decrease of cell migration and invasion capacity caused by CASC9 knockdown, providing sufficient evidence that LAMC2 was a target gene of CASC9.

Our study also demonstrated that CASC9 could activate the FAK-PI3K/Akt signaling pathways in ESCC. ECM components form focal adhesions with integrin family members on the cell membrane, and the most important fundamental adhesive element is the hemidesmosome (HD) [42], which is formed by the crucial integrin α6β4 and its ligand laminin-332 [43]. Upon ligation, focal adhesion kinase (FAK) is phosphorylated, thereby activating PI3K and Akt signaling pathways, as reported in breast and colon cancer, among others [7, 44, 45]. Our data indicated that both CASC9 and LAMC2 depletion attenuated the activation of FAK, PI3K, and Akt. Moreover, LAMC2 overexpression compromised the inactivation caused by CASC9 depletion. Many tumors present active signaling pathways, and the regulation networks between them are extremely complicated. In this study, we first detected FAK disorder seduced by LAMC2, and then we identified PI3K and Akt signaling pathways influenced by CASC9. Our research demonstrated that the FAK-PI3K/Akt signaling was enhanced in ESCC due to intensive ECM–integrin interactions regulated by CASC9. However, we cannot rule out other pathways regulated by CASC9 due to the limited detection methods, considering the significantly critical roles of CASC9 in ESCC.

LncRNAs can serve as guides or scaffolds for chromatin-modifying enzymes or transcriptional regulatory elements, which means that the regulatory function of lncRNAs is contingent upon which proteins they bind to [46]. CASC9 is located in a gene desert region with no protein-coding genes flanking nearby, and we discovered that CASC9 was located in both the cytoplasm and nucleus, suggesting that CASC9 could regulate gene expression in different ways. In this study, we discovered that CASC9 could bind with CBP to form an active complex that upregulates LAMC2 expression through increasing H3K27ac levels at LAMC2’s promoter. Most recently, a profile of eRNAs was reported to interact with CBP to promote gene expression through increasing histone acetylation [47]. Our study indicates that a lncRNA from a gene desert region harbors the capacity to bind with CBP. In our previous study, we demonstrated that CASC9 could recruit enhancer of zeste homolog2 (EZH2) to form a repressive complex, thereby downregulating the tumor suppressor gene PDCD4 expression through altering H3K27me3 levels at its promoter [22]. These findings demonstrate that CASC9 acts as a crucial oncogene in ESCC development in tumor growth and tumor metastasis. Emerging evidence demonstrates that lncRNAs can elevate or decrease the expression of different sets of genes to manipulate cancer development. One of the most widely studied lncRNAs in cancer biology, MALAT1, could regulate the expression of many tumor-related genes through interaction with different protein complexes [48]. It has been reported that MALAT1 associates with EZH2 and enhanced methylation of H3K27 to downregulate genes in renal cancer cells [49]; on the other hand, MALAT1 upregulates EGFL7 by altering the level of H3 histone acetylation at EGFL7 promoter in gastric cancer [50]. Therefore, from both CASC9 and MALAT1, we learn that lncRNAs are able to mediate assembly of multiple corepressors/coactivators and are capable of modifying the histone code, thereby coordinating two distinct, yet functionally related, chromatin-modifying activities. However, there are still several questions to be addressed as follows: Does CASC9 bind to CBP directly or indirectly? How does CASC9 identify and interact with precise genomic target sites? Does CASC9 have a function in the cytoplasm? We will focus on these issues in our future work.

**Fig. 8:**
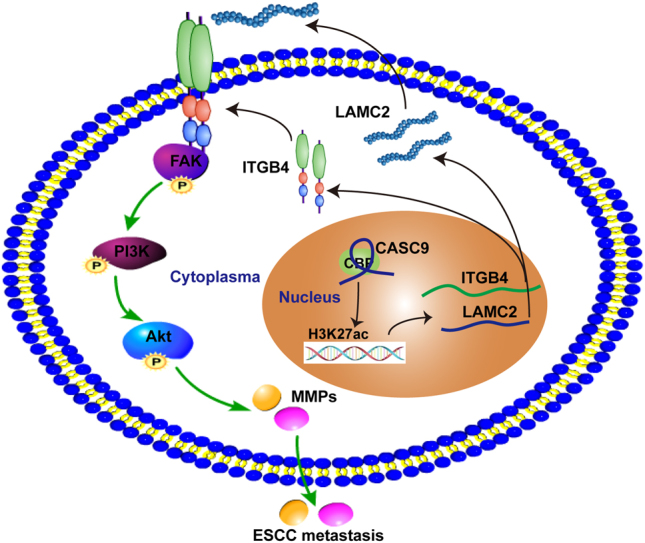
Model of CASC9 interaction with CBP and the signaling pathways involved in pro-metastatic function. **a** In the nucleus, CASC9 recruits CBP and modulates H3K27ac levels at the LAMC2 promoter, thereby facilitating gene transcription. **b** In the cytoplasm, LAMC2 is exported to the extracellular matrix and interacts with ITGB4 to activate FAK-PI3K/Akt signaling pathways, thereby promoting ESCC metastasis

In conclusion, our findings demonstrate the prognostic and pro-metastatic role of CASC9 in ESCC. We discovered that CASC9 stimulates gene expression via CBP-mediated histone acetylation and that its pro-metastatic role functions through the FAK-PI3K/Akt signaling pathways, mediated by LAMC2 (Fig. 8). Our findings underscore the crucial roles of CASC9 in ESCC metastasis and its potential prognostic and therapeutic value.

## Materials and methods

### Clinical tissues, RNA extraction, RT-qPCR analysis, and cell culture

Clinical tissue collection and storage, RNA extraction and detection, and cell culture were conducted, as described elsewhere [51]. The human normal esophageal epithelial cell line Het-1A and the ESCC cell lines EC109, KYSE150, and KYSE450 were identified by STR genotyping at Key Laboratory of Birth Defects and Reproductive Health (Chongqing, China) in 2015. The primers used in this study are listed in Supplementary Table 1. Gene expression was calculated using the 2^−△△Ct^ formula and normalized to GAPDH. Our research was approved by the Ethics Committee of the Third Military Medical University and executed in compliance with the Declaration of Helsinki.

### Pharmaceuticals

The histone acetyltransferase (HAT) inhibitor C646 was purchased from MCE (Medchem Express, USA). An appropriate number of cells was seeded in 6-well plates before media containing 20 μM of C646 was added to the culture. After 12 h, the cells were collected, and the total protein was isolated.

### Small interfering RNA (siRNA) synthesis, vector construction, and transfection

CASC9 siRNAs and shRNA were designed and synthesized by Shanghai GenePharma Company (Shanghai, China); LAMC2 siRNAs were designed and synthesized by Ribobio Company (Guangzhou, China); CBP and P300 siRNAs were synthesized by Oligobio Company (Beijing, China). The si-CASC9-2 siRNA and negative control were separately cloned into lentiviral vectors, named LV-CASC9 and LV-NC. The siRNA and shRNA sequences used in this study are listed in Supplementary Table 2. Full-length CASC9 (NR-103848.1) and LAMC2 (NM-005562.2) cDNAs were synthesized and cloned into the expression vector pcDNA3.1 (Invitrogen, USA). The antisense CASC9 (AS-CASC9) sequence was generated from CASC9 expression vector and then cloned into a pcDNA3.1 vector to generate the AS-CASC9 expression vector. All PCR products were verified with DNA sequencing.

Cell transfection was performed using Lipofectamine 2000 reagent (Invitrogen, USA). To obtain the CASC9 stable knockdown cell line, KYSE150 cells were infected with LV-CASC9 and LV-NC in the presence of 6 µg/ml polybrene and selected with 10 µg/ml puromycin.

### Migration and invasion assays

Transfected cells were harvested for invasion and migration assays. For the invasion assay, Matrigel (Corning, USA) was diluted with serum-free RPMI-1640 media and then plated into the upper chamber of an insert with an 8-μm pore size (Corning, USA). Total of 5 × 10^4^ and 1 × 10^5^ cells were seeded into the upper chamber for migration and invasion assays, and media with 20% (FBS) were added into the lower chamber. After incubation for 36 h, cells remaining in the upper chamber were wiped off, and cells on the lower chamber were fixed with 4% paraformaldehyde and stained with crystal violet. The number of cells that migrated or invaded were counted in three different fields with a microscope (Olympus, Tokyo, Japan).

### Wound healing assay

Transfected cells were cultured in serum-free RPMI-1640 media and scratched using sterile 100-μl pipette tips at 80–90% confluence. A microscopic observation was recorded every 12 h immediately after the scratch with microscope (Olympus, Tokyo, Japan). Wound distance was measured and calculated as a percentage of the distance at 0 h.

### In vivo assay

Male athymic BALB/c nude mice (5 weeks) were purchased and maintained in laminar flow cabinets under specific pathogen-free conditions. Mice were divided randomly into two groups, and 1 × 10^6^ cells were injected into the tail vein of nude mice, which were subsequently killed 8 weeks later. The lungs were anatomized and photographed. Then, the tissues were fixed with 4% paraformaldehyde and paraffin embedded (FFPE) before being analyzed with hematoxylin and eosin (HE) staining to confirm pathology. Our research was in strict accordance with the Care and Use of Laboratory Animals of the National Institutes of Health.

### Western blot assay

Clinical tissues or cultured cells were lysed with either T-PER^TM^ Tissue Protein Extraction Reagent (Thermo, USA) or RIPA Lysis Buffer (Thermo, USA) on ice, supplemented with protease and phosphatase inhibitor reagents (Thermo, USA). Then, 20 μg of total protein was separated and transferred to a 0.22-μm PVDF membrane (Millipore, USA). PVDF membranes were then blocked in 3–8% skim milk for 1–3 h at room temperature. Primary antibodies were incubated overnight at 4 °C; HRP-conjugated secondary antibodies were incubated for 1 h at room temperature. The protein bands were detected using chemiluminescence with imaging system (Bio-Rad, USA). An anti-GAPDH antibody (#KC-5G5, KangChen, China) was used as an internal reference; anti-Laminin-5 (γ2 chain) (MAB19562) was from Millipore (Millipore, USA); anti-histone H3 (D2B12), anti-ITGB4 (#4707), FAK Antibody Sampler Kit (#9330), anti-PI3K Kinase p110α (#4249), anti-Phospho-PI3 Kinase p85 (Tyr458)/p55(Tyr199) (#4228), anti-AKT (pan) (#4691), and anti-phospho-Akt (Ser473) (#4060) were from Cell Signaling Technology (CST, USA); anti-MMP10 antibody (ab199688) and anti-MMP13 (ab39021), antibody anti-KAT3A/CBP (ab2832), and anti-histone H3 (acetyl K27) (ab4729) were from Abcam (Abcam, UK).

### Immunofluorescence (IF) and immunohistochemical (IHC) assays

For IF, cells were seeded onto sterile slides after transfection. When cell confluence reached 80–90%, cells were fixed with 4% paraformaldehyde for 30 min, permeabilized with 0.3% Triton X-100 for 30 min and then blocked with 10% normal goat serum for 30 min. Cells were then incubated with anti-ITGB4 (#4707, CST) or anti-laminin-5 (γ2 chain) (MAB19562, Millipore) at a 1:50 dilution overnight at 4 °C, followed by further incubation at room temperature for 1 h with rabbit IgG (Alexa Fluor 546, green) (A11010, Thermo, USA) or mouse IgG (Alexa Fluor 647, red) (A0473, Beyotime, China) at 1:500; nuclear DNA was labeled in blue with DAPI.

For IHC, FFPE tissue sections were first baked and deparaffinized. Heat or enzymatic-induced antigen retrieval was performed. Sections were then subjected to incubation with either the Ki67 (ab15580, Abcam) or CK5 (ab52635, Abcam) antibody followed by secondary antibody incubation.

### RNA fluorescent in situ hybridization (FISH)

FISH assay was performed using Ribo^TM^ Fluorescent In Situ Hybridization Kit (Ribobio Company, China). CASC9 and U6 probes were designed and synthesized by Ribobio Company and labeled with Cy3 fluorescent dye. Briefly, cells were seeded onto sterile slides until cell confluence reached 60–70%. Cells were then fixed with 4% paraformaldehyde and permeabilized with 0.5% Triton X-100 for 30 min. Next, cells were blocked with pre-hybridization buffer/blocking solution for 30 min at 37 °C with gentle rocking, followed by incubation in 0.5 µg/ml probe/hybridization buffer at 37 °C for 16–20 h with gently rocking. The next day, cells were washed with saline sodium citrate (SSC) buffer solution and stained with DAPI for 10 min. Finally, the slides were removed from the plate and mounted with 50% glycerol in PBS. Fluorescence detection was performed with a confocal laser-scanning microscope (LSM 780, Zeiss).

### Dual-luciferase reporter assay

The pGL3-basic and PRL-TK vectors were purchased from Promega (Promega, USA). Sequences containing the LAMC2 promoter region were cloned into Mlu and Xho1 restriction sites of the pGL3-basic vector to generate the pGL3-LAMC2 reporter construct. All the constructs were confirmed by sequencing. Cells were co-transfected with a reporter construct (pGL3-basic plasmid or pGL3-LAMC2 plasmid) and siRNA or negative control. Forty-eight hours later, luciferase activity was measured using the Dual-Luciferase® Reporter Assay System (E1910, Promega).

### RNA–protein pull-down assay

Vectors were transcribed in vitro to generate full-length CASC9 and AS-CASC9 RNA using TranscriptAid T7 High Yield Transcription Kit (#K0441, Thermo, USA). Then, RNAs were labeled with desthiobiotinylate using Pierce^TM^ RNA 3′end Desthiobiotinylation Kit (#20163, Thermo, USA). The labeling efficiency was tested with Chemiluminescent Nucleic Acid Detection Module (#89880, Thermo, USA). An RNA–protein pull-down assay was performed using Pierce^TM^ Magnetic RNA-protein Pull Down Kit (#20164, Thermo, USA). In brief, 50 pmol labeled CASC9 and AS-CASC9 RNA bind to streptavidin magnetic beads, and 80 μg of protein was used to bind RNA. The RNA–protein beads mixture was incubated for 1 h at 4 °C with rotation. Finally, the RNA-binding protein was eluted and analyzed by western blot.

### RNA-binding protein immunoprecipitation (RIP) assay

RIP assay was performed using the EZ-Magna RIP RNA-Binding Protein Immunoprecipitation Kit (Cat. #17-701, Millipore, USA). Cells were cultured in 75-cm^2^ flasks to 80–90% confluence. Then, cells were harvested by scraping. One RIP reaction required 100 μl of cell lysate from ~2.0 × 10^7^ cells. Next, 5 μg of purified antibodies and corresponding IgG was added to the 100-μl cell lysate, and the mixture was incubated with rotation overnight at 4 °C. Anti-SNRNP70 (Cat. #CS203216, Millipore), anti-KAT3A/CBP (ab2832, Abcam) and normal rabbit IgG (Cat. #PP64B, Millipore) were used for RIP assay. The immunoprecipitated RNA was purified and analyzed with RT-qPCR, and the results were calculated as a percentage of input RNA using the following formula: Percent input = 2% × 2 (C[T] 2% input sample − C[T] IP sample).

### Chromatin immunoprecipitation (ChIP) assay

ChIP was performed using the SimpleChIP® Enzymatic Chromatin IP Kit (#9003, CST, USA). First, cells were harvested with protein cross linked to DNA using 1% formaldehyde. Then, chromatin was digested to lengths of ~100–900 bp using Micrococcal Nuclease. Next, 5–10 μg of digested and cross-linked chromatin combined with 2 μg of antibody was used per immunoprecipitation. Anti-KAT3A/CBP (ab2832, Abcam) and anti-histone H3 (acetyl K27) (ab4729, Abcam) were employed in ChIP assay, with goat anti-rabbit IgG used as a negative control. The retrieved DNA was quantified by real-time qPCR analysis with the specific primers listed in Supplementary Table 1. The results were calculated as a percentage of input DNA using the same formula as for the RNA pull-down.

### Statistical analysis

Experimental data are presented as the mean ± SE from three independent experiments performed in triplicate. All statistical analyses were performed using SPSS 16.0 software (SPSS, USA). The significant differences between groups were calculated by Student’s *t* test, Chi-square test or one-way ANOVA, as appropriate. Survival curves were constructed by the Kaplan–Meier method, and a log-rank test was used for comparison. Two-sided *P* values were calculated, and a *P* < 0.05 was considered to be statistically significant.

## Electronic supplementary material

supplementary materials
