# Supplementary material for: LncRNA CASC9 promotes esophageal squamous cell carcinoma metastasis through upregulating LAMC2 expression by interacting with the CREB-binding protein
Source: Cell Death Differ. 2018 Mar 6;25(11):1980–95. doi: 10.1038/s41418-018-0084-9 (PMC6219493; doi:10.1038/s41418-018-0084-9)

Supplementary Figure 1


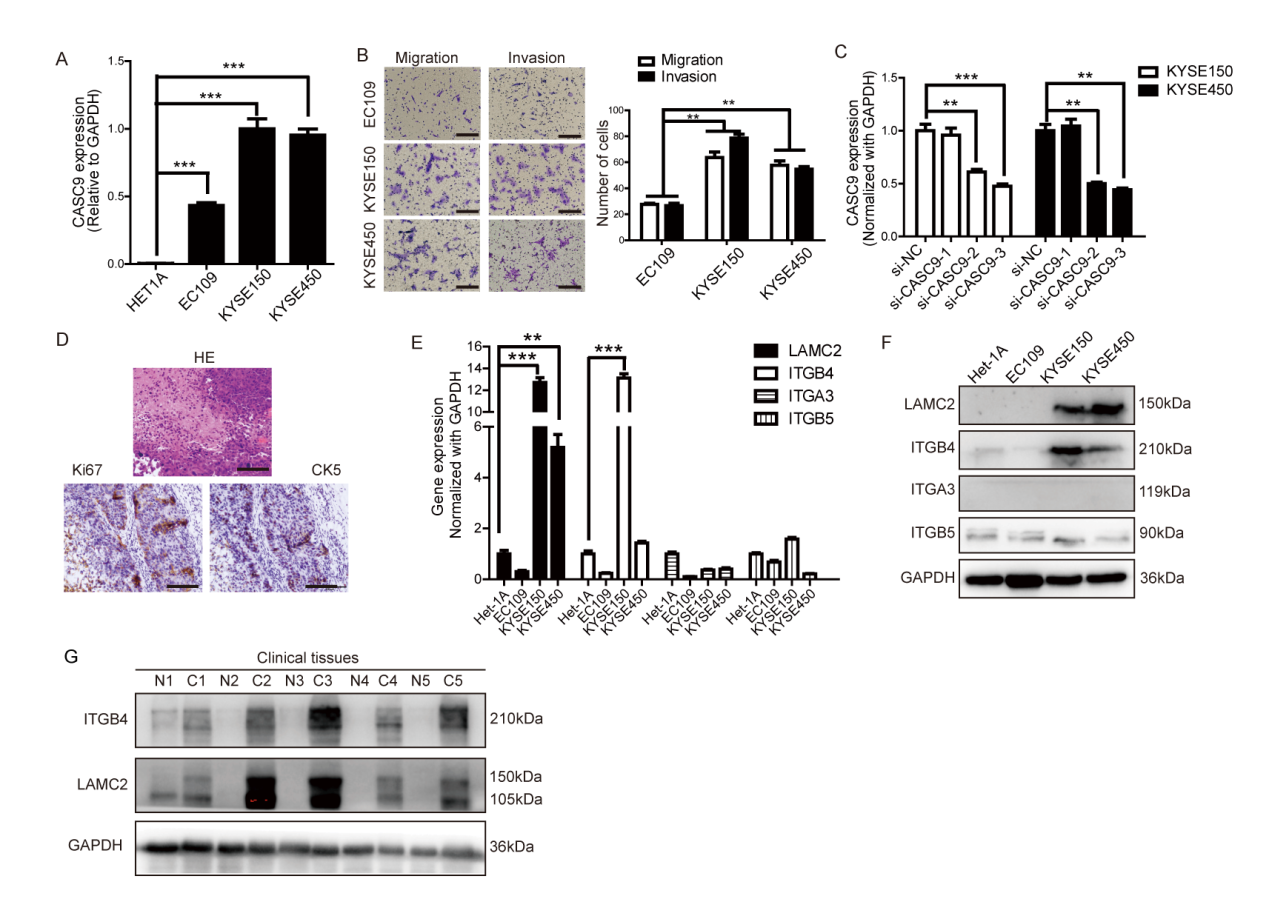


Figure S1. (A) CASC9 expression was higher in EC109, KYSE150 and KYSE450 than Het-1A. (B) The expression of CASC9 was positively correlated with the migration and invasion ability of ESCC cell lines. (C) The CASC9 interference efficiency in KYSE150 and KYSE450 using 3 different siRNAs and negative control detected by RT-qPCR. (D) Representative images of HE and IHC staining of metastasis from other positions. Original magnification, ×400. Scale bars, 100um. (E, F) Western blot and RT-qPCR analysis of the expression of LAMC2, ITGB4, ITGA3 and ITGB5 in ESCC cell lines and normal esophageal epithelia cell line. (G) Representative images of western blot analysis of LAMC2 and ITGB4 expression in ESCC tissues and normal tissues. N represents for normal tissues, C represents for cancer tissues. *P<0.05, **P<0.01, ***P<0.001.

Supplementary Figure 2


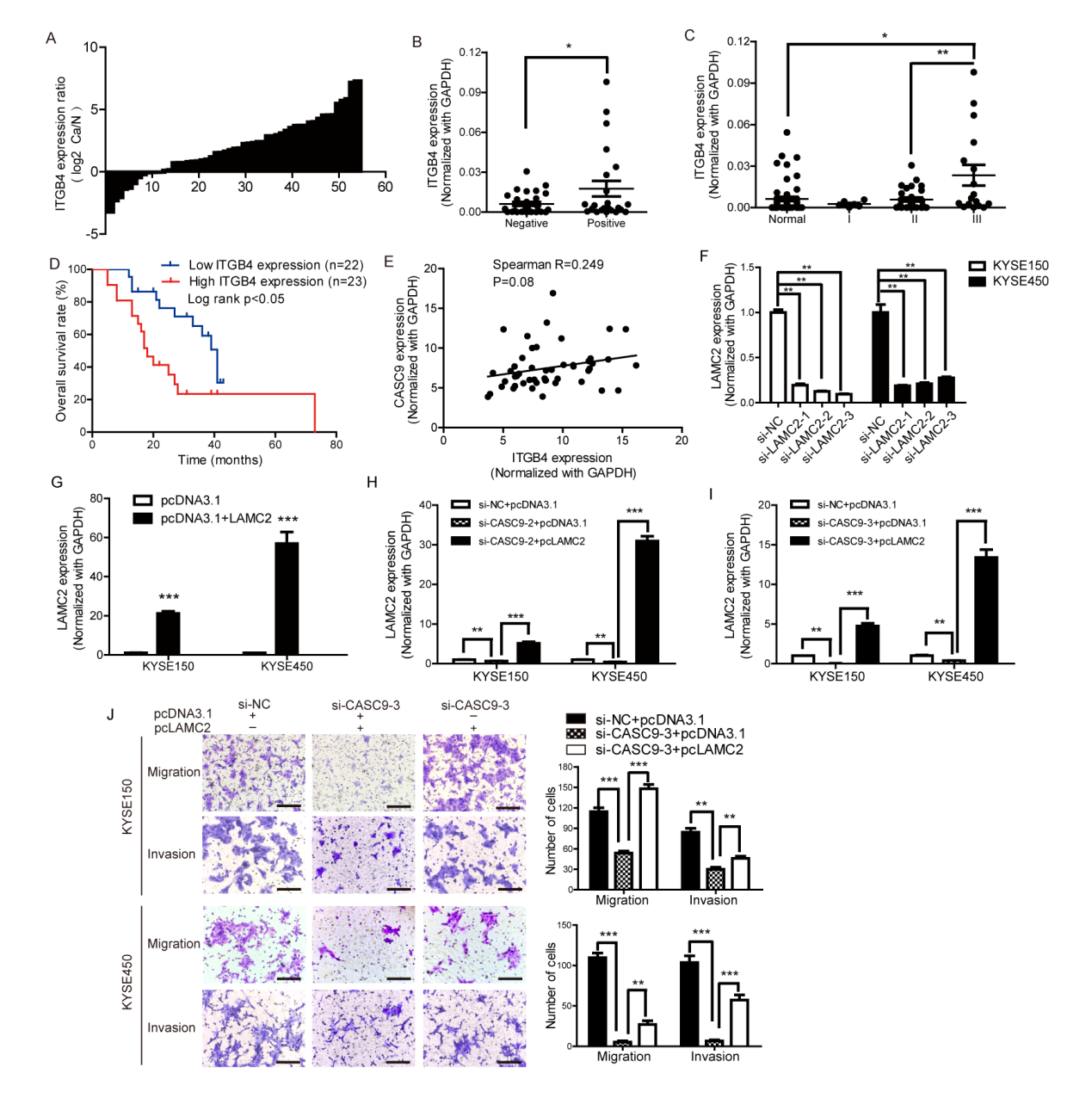


Figure S2. (A) Fold change of ITGB4 expression between ESCC tissues and normal tissues normalized by log2 using RT-qPCR. (B) ESCC patients with lymphnode metastasis presented higher ITGB4 expression. (C) The correlation between ITGB4 expression and ESCC TNM stage. (D) Kaplan-Meier analysis of 37 patients indicated that ITGB4 expression had no impact on overall survive rate. (E) Correlation analysis of ITGB4 and CASC9 expression in ESCC tissues. Axes value are transformed by -Log2 of CASC9 expression. (F) The LAMC2 interference efficiency using 3 different siRNAs and negative control detected by RT-qPCR. (G) RT-qPCR analysis showed the efficiency of LAMC2 transfected with LAMC2 expression plasmid and pcDNA3.1. (H, I) RT-qPCR analysis showed LAMC2 expression in KYSE150 and KYSE450 during rescue experiments. (J) The rescue experiment with Transwell assays was performed in KYSE150 and KYSE450 cells co-transfected with si-NC or si-CASC9-3 and pcDNA3.1 or pcLAMC2. Original magnification, ×100. Scale bars, 200 µm. *P<0.05, **P<0.01, ***P<0.001.

Supplementary Figure 3


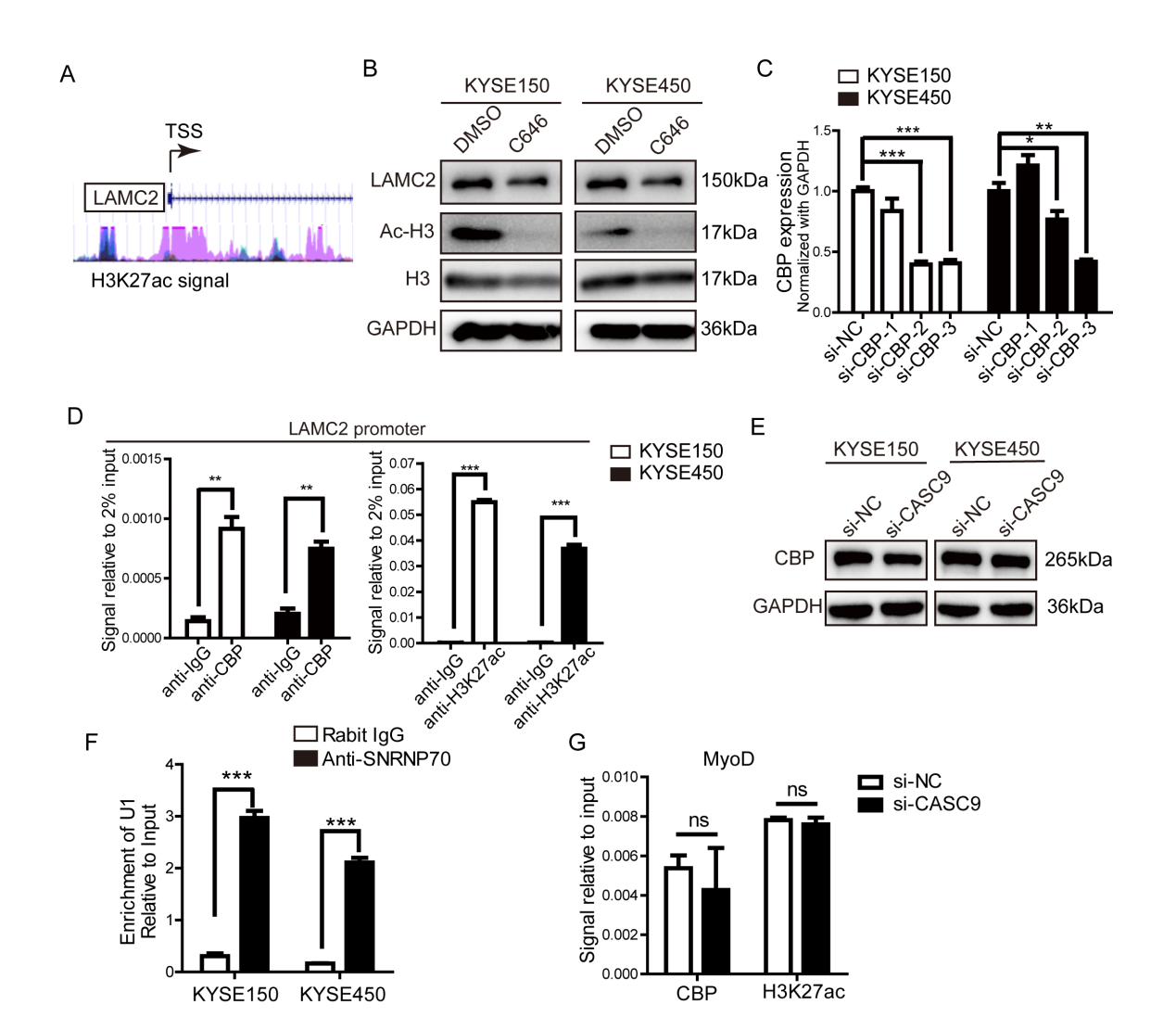


Figure S3. (A) The H3K27ac signal of LAMC2 promoter. (B) Western blot analysis of LAMC2 expression after C646 treatment in KYSE150 and KYSE450. C646 treatment: 20μM, 12h. (C) The CBP interference efficiency using 3 different siRNAs and negative control detected by qRT-PCR. (D) ChIP-qPCR analysis of CBP and H3K27ac enrichment at LAMC2 promoter. (E) Western blot analysis indicated that CASC9 inhibition had no affect on CBP expression. (F) Positive control of RIP assay using anti-SNRNP70 antibody. (G) Negative control of ChIP assay using anti-MyoD antibody. *P<0.05, **P<0.01, ***P<0.001.

Supplementary Table 1: Sequences of Primers

| qRT-PCR Primers | Sequences 5’-3’ | PCR product length |
| --- | --- | --- |
| GAPDH-F | GGGAGCCAAAAGGGTCATCA | 203bp |
| GAPDH-R | TGATGGCATGGACTGTGGTC |  |
| CASC9-F | TTGGTCAGCCACATTCATGGT | 119bp |
| CASC9-R | AGTGCCAATGACTCTCCAGC |  |
| LAMC2-F | GCCTTTTGGCACCTGTATTC | 92bp |
| LAMC2-R | CAGGATTCTCATCCCCTGAA |  |
| FLNA-F | AGCCTCCACGAGACATCATC | 310bp |
| FLNA-R | CCAGTGTGTACTCCCCCTTG |  |
| GRB2-F | ATTCCTGCGGGACATAGAACA | 196bp |
| GRB2-R | GGTGACATAATTGCGGGGAAAC |  |
| TNC-F | TGG GAGATCATCTTCCGGAAT | 152bp |
| TNC-R | CCC CGGGTATTGTTTTTCACT |  |
| THBS1-F | AAGAAGCTCTCCTGGCAACC | 131bp |
| THBSI-R | GAAGACGCTTTGGATGGGGA |  |
| ITGA3-F | TATTGAGGACATGTGGCTTG | 92bp |
| ITGA3-R | ACAGCACCTGGGTGTAGC |  |
| ITGB4-F | CATGAGGCCTGAGAAGCTGA | 139bp |
| ITGB4-R | ATCCAGGTTGCCTGAGATCC |  |
| ITGB5-F | CAGGTGGAGGACTATCCTGTG | 190bp |
| ITGB5-R | GTGCCGTGTAGGAGAAAGGAG |  |
| PIK3CA-F | CGTTTCTGCTTTGGGACAAC | 100bp |
| PIK3CA-R | CCTGATGATGGTCGTGGAG |  |
| VEGFC-F | GCTTCTTCTCTGTGGCGTGT | 177bp |
| VEGFC-R | ACACAGACCGTAACTGCTCC |  |
| CBP-F | GTGCTGGCTGAGACCCTAAC | 125bp |
| CBP-R | GGCTGTCCAAATGGACTTGT |  |
| P300-F | CAATGAGATCCAAGGGGAGA | 151bp |
| P300-R | ATGCATCTTTCTTCCGCACT |  |
| MMP10-F | ATCAACCTTAGGCTCAACT | 199bp |
| MMP10-R | TCCAAGAGGCATCCATAC |  |
| MMP13-F | GCCATCGTGAAGTCTGGT | 170bp |
| MMP13-R | AATACAATGTTTTCCCTCG |  |
| ChIP-qPCR primers | Sequences 5’-3’ | PCR product length |
| LAMC2-F | CTCCGGGGAATCTCGCACA | 144bp |
| LAMC2-R | CACAAACCGGGCTGGAAAATC |  |
| MyoD-F | ATGGACTACAGCGGCCC | 74bp |
| MyoD-R | GGCGCCTCGTTGTAGTAGG |  |

Supplementary Table 2: siRNA sequences

| CASC9 siRNAs | Sequences 5’-3’ | siRNA length |
| --- | --- | --- |
| si- CASC9-1 | GGGCA UUGAG AAGUU AGAATT | 21nt |
| si- CASC9-2/sh-CASC9 | GCCUG UGAUA GCAGA ACAATT | 21nt |
| si- CASC9-3 | GGACU CAUAU UACCA GUCUTT | 21nt |
| LAMC2 siRNAs | Sequences 5’-3’ | siRNA length |
| si- LAMC2-1 | GAAGC TTCCT TGGGA AACA | 19nt |
| si- LAMC2-1 | GTCAA AGCCT GTCCT TTGA | 19nt |
| si- LAMC2-1 | TCGGG AACTT CACAG ACAA | 19nt |
| CBP siRNAs | Sequences 5’-3’ | siRNA length |
| si-CBP-1 | CGGCA CAGCC TCTCA GTCA | 19nt |
| si-CBP-2 | GGAGC CATCT AGTGC ATAA | 19nt |
| si-CBP-3 | GGAAC TAGAA CAAGA AGAA | 19nt |


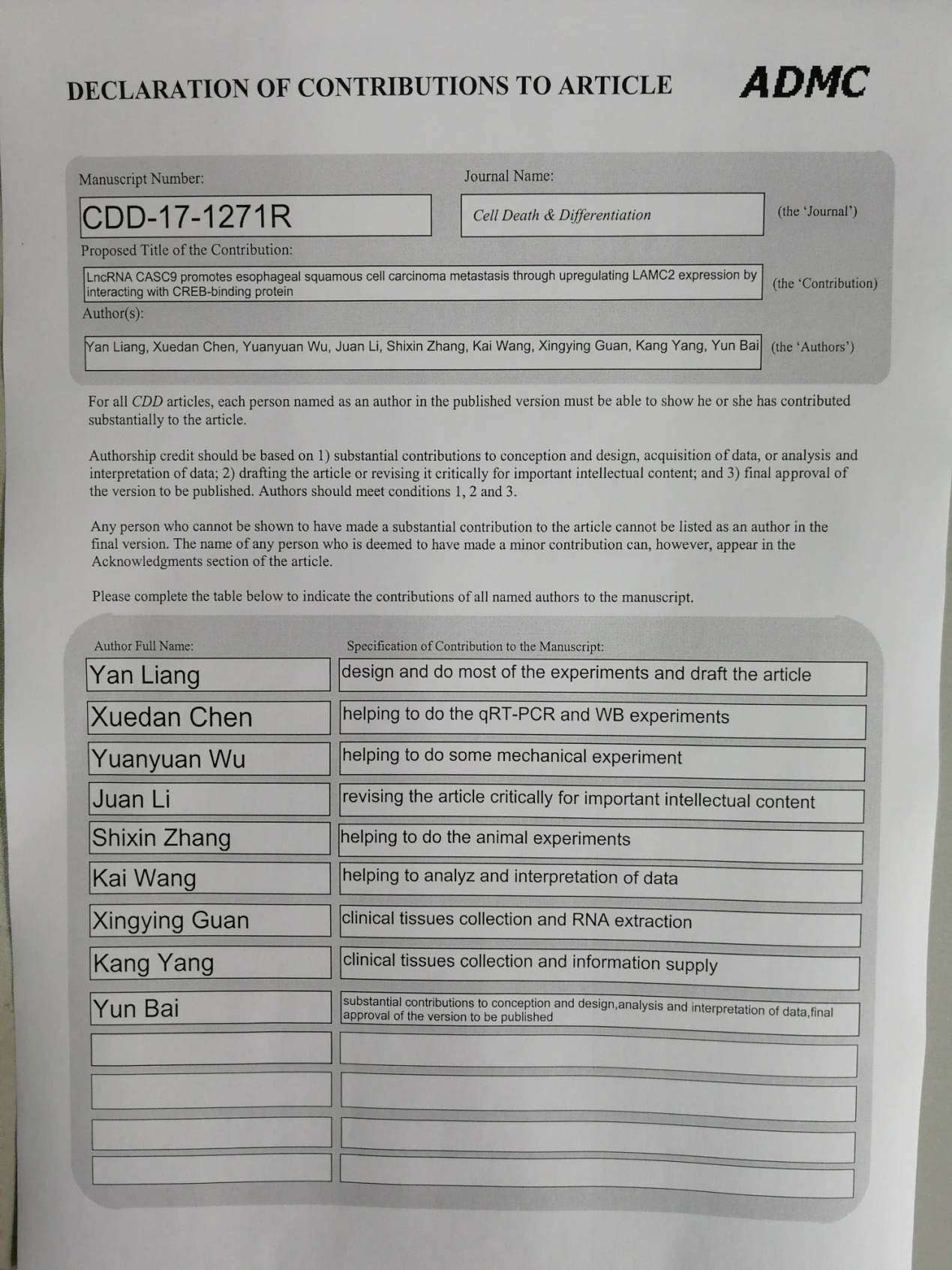


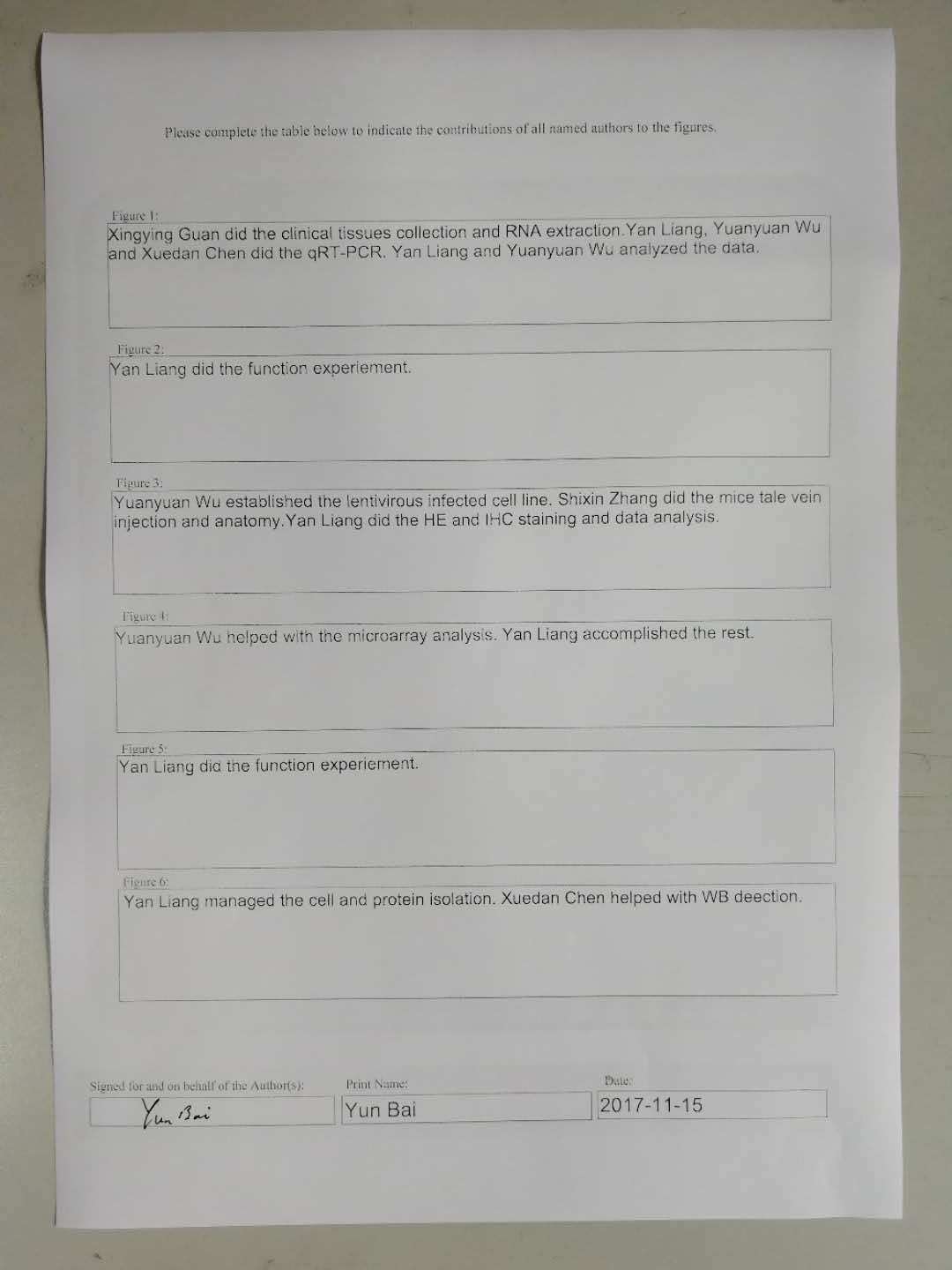

Supplement: Supplementary file 1 — supplementary materials [file 41418_2018_84_MOESM1_ESM.docx]
